# From individual coping to collective resilience: reframing mental health responses to global crises through geopsychiatry

**DOI:** 10.3389/fpubh.2026.1930193

**Published:** 2026-08-05

**Authors:** Julio Torales, Lairtes Chaves Rodrigues Filho, Gladys Estigarribia, Iván Barrios, Marcelo O’Higgins, Antonio Ventriglio, João Mauricio Castaldelli-Maia

**Affiliations:** 1 Facultad de Ciencias Médicas, Grupo de Investigación sobre Epidemiología de los Trastornos Mentales, Psicopatología y Neurociencias, Universidad Nacional de Asunción, San Lorenzo, Paraguay; 2 Vicerrectoría de Investigación y Postgrado, Universidad de Los Lagos, Osorno, Chile; 3 Facultad de Ciencias de la Salud, Grupo de Investigación en Comunicación, Salud Mental y Humanidades Médicas, Universidad Interamericana, Pedro Juan Caballero, Paraguay; 4 Instituto Regional de Investigación en Salud, Universidad Nacional de Caaguazú, Coronel Oviedo, Paraguay; 5 Facultad de Ciencias Médicas, Filial Santa Rosa del Aguaray, Cátedra de Bioestadística, Universidad Nacional de Asunción, Santa Rosa del Aguaray, Paraguay; 6 Department of Clinical and Experimental Medicine, University of Foggia, Foggia, Italy; 7 Department of Psychiatry, University of São Paulo, São Paulo, SP, Brazil

**Keywords:** collective coping, collective resilience, emotional engagement, geopsychiatry, global crises, public mental health, social determinants of mental health

## Abstract

Global crises are reshaping the conditions under which mental health is produced, threatened, protected, and restored. Pandemics, climate change, armed conflict, forced migration, political instability, economic insecurity, disasters, and digital disruption do not affect mental health only through individual stress reactions or discrete traumatic exposures. They also transform the social, territorial, institutional, ecological, and symbolic environments in which people interpret threat, seek support, access care, maintain hope, and imagine the future. This Conceptual Analysis reframes mental health responses to global crises by moving from an individual-centered model of coping toward a geopsychiatric understanding of collective resilience. We synthesize and extend published conceptual and empirical literature on global mental health, social determinants, disaster psychiatry, community resilience, emotional engagement, collective coping, climate-related distress, migration, structural violence, syndemics, digital environments, and geopsychiatry. We argue that resilience in global crises is not only a psychological capacity located within individuals, but also a collective, territorial, cultural, institutional, digital, and political process. The proposed geopsychiatric model links global crises to mental health outcomes through multilevel pathways involving individuals, relationships, communities, territories, institutions, digital ecosystems, and planetary systems. Emotional engagement, social meaning, institutional trust, access to resources, recognition, and collective efficacy are presented as mediating processes through which crises may either fragment communities or mobilize collective protection. This framework has implications for public mental health, policy, and care systems, suggesting that effective crisis responses should strengthen community supports, trusted communication, social protection, culturally meaningful care, digital responsibility, and climate- and crisis-resilient health systems. Collective resilience should therefore be understood not as a private duty, but as a geopsychiatric condition shaped by relationships between people, places, institutions, ecologies, and futures.

## 1 Introduction

Global crises are increasingly shaping the conditions under which mental health is produced, threatened, protected, and restored. Pandemics, climate change, armed conflict, forced migration, political instability, disasters, economic insecurity, and digital disruption do not affect psychological well-being only through discrete traumatic exposures or individual stress reactions. They also transform the social, territorial, institutional, ecological, and symbolic environments in which people interpret threat, seek support, maintain hope, access care, and imagine the future. Contemporary public mental health therefore faces a conceptual challenge: how should resilience be understood when the sources of distress are not merely personal adversities, but large-scale, prolonged, and interconnected disruptions of everyday life? (1–6).

The global mental health field has already moved beyond narrow biomedical models by emphasizing social determinants, sustainable development, equity, human rights, and community-based care (1–3). These perspectives are especially relevant in times of global crisis, when uncertainty, loss, displacement, institutional fragility, and social fragmentation accumulate across multiple ecological levels. During the COVID-19 pandemic, global estimates suggested substantial increases in depressive and anxiety disorders, reflecting not only infection-related fears but also bereavement, social restrictions, economic insecurity, and service disruption (4). In conflict-affected settings, systematic evidence used to generate WHO prevalence estimates has shown a high burden of depression, anxiety, post-traumatic stress disorder, bipolar disorder, and schizophrenia (5). Climate change has also been associated with psychological distress, including anxiety, depression, trauma-related symptoms, suicide-related outcomes, solastalgia, ecological grief, climate anxiety, and functional disruption related to extreme weather events, displacement, livelihood insecurity, and anticipatory concern about environmental futures (6–8). Taken together, these bodies of evidence suggest that global crises are not isolated external events acting on otherwise stable individuals; rather, they alter the social and ecological contexts that sustain mental health.

Despite this broader evidence base, much clinical and public discourse continues to frame coping and resilience primarily as individual attributes. This emphasis is understandable, as individual-level constructs such as emotional regulation, adaptive coping, self-efficacy, psychological flexibility, and hope remain clinically useful. However, an exclusively individual-centered model risks obscuring the extent to which people’s capacity to cope depends on conditions outside the person, including social cohesion, safety, housing, livelihood, institutional trust, political stability, cultural continuity, access to care, and credible information. In crisis contexts, asking individuals to be resilient without addressing the environments that generate vulnerability may inadvertently shift responsibility from systems to persons. A more adequate framework must therefore consider how resilience is distributed, enabled, constrained, and sometimes undermined by collective and territorial conditions.

Evidence from disaster mental health, community psychology, and social resilience research supports this broader view. Hobfoll et al. (9) identified five empirically supported principles for intervention after mass trauma: safety, calming, self- and community efficacy, connectedness, and hope. Norris and colleagues conceptualized community resilience as a process linking adaptive capacities to positive adaptation after disturbance, emphasizing economic development, social capital, information and communication, and community competence (10). Ungar’s social ecological approach similarly argues that resilience depends on the availability and accessibility of culturally meaningful resources, not only on individual traits (11). Indigenous and community mental health scholarship has also shown that resilience may be shaped by collective identity, cultural continuity, historical context, and community-level resources (12). Recent work on social resilience and collective mental health further emphasizes collective action, community bonds, social capital, shared values, equitable access to resources, and the capacity of communities to adapt, recover, and protect mental health under adversity (13).

This article argues that geopsychiatry offers a useful conceptual framework for integrating these insights. Geopsychiatry has been described as a field concerned with the interface between geography and psychiatry, including the mental health effects of climate change, disasters, globalization, population movement, urbanization, and other geopolitical conditions (14). Related work has emphasized that mental health is influenced by forces operating beyond the clinic, including governance, international relations, conflict, migration, inequality, and public policy (15). More recent geopsychiatric writing has connected climate change, migration, and global mental health by highlighting how environmental, political, economic, and territorial forces shape vulnerability, adaptation, and psychiatric outcomes (7, 16). Empirical work on geopsychiatry education also suggests that, despite growing recognition of the field’s importance, psychiatric training remains insufficiently equipped to prepare clinicians for environmental, sociopolitical, and geopolitical determinants of mental health (17). Geopsychiatry is therefore not simply the study of “place” as a background variable, but a way of examining how territory, power, ecology, culture, institutions, mobility, and policy shape mental suffering and recovery.

A geopsychiatric approach also helps clarify why emotional engagement in global crises should be understood as a public mental health process rather than a purely private psychological reaction. Crises are interpreted through social narratives, media ecosystems, political communication, cultural memory, institutional credibility, and community experience. Threat-oriented geopolitical rhetoric, for example, may influence population mental health through symbolic threat, perceived uncertainty, loss of control, media amplification, and differential vulnerability across social groups (18). Such processes show that fear, grief, anger, hopelessness, solidarity, trust, and collective efficacy are shaped by the environments in which people receive information, evaluate risk, and locate themselves within a shared future.

For the purposes of this Conceptual Analysis, emotional engagement refers to the ways individuals and communities perceive, interpret, share, regulate, and mobilize emotions in response to crisis. This definition is grounded in evidence that emotions are socially shared, collectively shaped, and capable of influencing interpersonal regulation, group meaning-making, and public responses to threat (19, 20). Collective coping refers to the practices through which groups, communities, institutions, and care systems respond to shared threats, including mutual aid, communication, rituals, solidarity, advocacy, and community-based support. These practices are closely related to social identity, collective efficacy, and collective action, which can shape how groups interpret adversity and mobilize supportive responses (9, 21, 22). Community resilience refers not only to recovery after adversity, but also to the capacity of communities to preserve meaning, sustain relationships, protect vulnerable members, adapt institutions, and transform harmful conditions (10–13, 22). A geopsychiatric lens integrates these processes by asking where crises occur, who is most exposed, which institutions are trusted, how digital and physical spaces shape emotional responses, and what forms of community care are locally available (7, 14, 16–18).

However, existing approaches often remain fragmented. Clinical models tend to emphasize symptoms, coping strategies, and individual treatment; disaster and humanitarian frameworks focus on preparedness, psychosocial support, and service delivery; climate and migration literatures highlight environmental and territorial vulnerability; and social resilience scholarship emphasizes networks, trust, and collective efficacy. Each of these perspectives is valuable, but none fully captures how emotional engagement, collective coping, territorial exposure, institutional trust, digital environments, and systems of care interact in global crises. This fragmentation matters because public mental health responses may remain either too individualized, too service-centered, or too detached from the social and political conditions that shape distress. A geopsychiatric framework can help integrate these dimensions by locating mental health within the relationships between people, places, institutions, ecologies, and futures.

The aim of this Conceptual Analysis is therefore to reframe mental health responses to global crises by moving from an individual-centered model of coping toward a geopsychiatric understanding of collective resilience. We do not present original data. Instead, we synthesize and extend published conceptual and empirical literature to clarify key concepts and propose an integrative model linking emotional engagement, collective coping, territorial vulnerability, institutional trust, digital environments, and systems of care. The central claim is that resilience in global crises is not only a psychological capacity located within individuals, but also a collective, territorial, cultural, institutional, digital, and political process. By developing this argument, the article seeks to support public mental health responses that strengthen communities, rebuild trust, address structural vulnerability, and organize care so that people are not left to cope alone.

## 2 The limits of individual-centered resilience in global crises

The language of coping and resilience has become central to contemporary mental health practice, disaster response, humanitarian care, and public health. In clinical psychology and psychiatry, coping has often been understood through transactional models of stress, in which individuals appraise stressors, evaluate available resources, and deploy cognitive or behavioral strategies to manage demands perceived as exceeding their capacities (23). Similarly, resilience has been conceptualized as the capacity to maintain or regain adaptive functioning in the face of adversity, trauma, stress, or threat (24–28). These concepts remain clinically useful because they emphasize agency, adaptation, recovery, and protective resources.

However, they become limited when applied to global crises without sufficient attention to context. Resilience can easily be reduced to an individual trait or a personal capacity to “bounce back.” Such a view may be useful in some clinical or research settings, but it is insufficient when the stressor is not a discrete life event but a prolonged, structural, and collective disruption. Climate change, pandemics, armed conflict, forced migration, political instability, economic insecurity, and digital misinformation are not simply adversities encountered by individuals. They reshape the environments in which coping occurs. Under these circumstances, resilience cannot be understood only as personal toughness, emotional regulation, optimism, or self-efficacy (10, 11, 13, 29).

A strictly individual-centered model also risks detaching psychological adaptation from the distribution of risk. In global crises, vulnerability is rarely random. Exposure to heat, displacement, violence, unemployment, food insecurity, institutional neglect, or disrupted healthcare is structured by geography, class, gender, ethnicity, disability, migration status, age, and political conditions (1–7). Individuals may differ in coping style, but their capacity to cope is shaped by whether they have housing, income, family support, community safety, access to medicines, legal protection, credible information, and functioning services. Coping is therefore never purely intrapsychic; it is enabled or constrained by material, social, institutional, and territorial conditions.

An intersectional perspective further clarifies why vulnerability cannot be understood through isolated social categories. Building on Crenshaw’s work, intersectionality draws attention to how social positions and systems of power (including gender, race and ethnicity, socioeconomic position, age, disability, migration status, and geographical location) interact to produce distinct, rather than merely additive, patterns of exposure, recognition, access to protection, and mental health consequences (30). During global crises, the same event may therefore generate qualitatively different risks for people situated at the intersection of multiple forms of disadvantage. A geopsychiatric analysis should consequently ask not only where vulnerability is concentrated, but also how territorial, institutional, historical, and sociopolitical forces intersect in particular lives and communities.

This broader contextualization matters because resilience discourse can unintentionally shift responsibility from systems to individuals. When institutions emphasize resilience without addressing the conditions that produce vulnerability, resilience may become a moral expectation rather than a protective framework. Individuals and communities may be asked to adapt to intolerable circumstances while the political, environmental, or institutional sources of distress remain unchanged. The problem is not resilience itself, but a decontextualized use of the concept that avoids asking who is expected to be resilient, under what conditions, with which resources, and at whose expense. Contextual and social ecological approaches provide an important corrective by locating resilience within culturally meaningful resources, relationships, institutions, and material environments (10, 11, 13, 29).

The limitations of individual-centered resilience are also evident in disaster and mass trauma contexts. Hobfoll and colleagues’ five essential principles after mass trauma (safety, calming, self- and community efficacy, connectedness, and hope) already imply that post-crisis recovery depends on more than individual coping skills (9). Safety requires protection from ongoing threat; calming depends partly on trustworthy information and social containment; efficacy involves both personal and collective agency; connectedness requires social networks; and hope is sustained by credible pathways toward recovery. These principles point toward an ecological and collective understanding of resilience.

Community resilience research provides a further corrective to individualism. Norris and colleagues described community resilience not as a fixed trait, but as a process through which adaptive capacities support positive adaptation after disturbance (10). Ungar’s social ecological model similarly argues that resilience depends on the availability and accessibility of culturally meaningful resources, while Panter-Brick has emphasized that resilience research must attend to culture, political economy, inequality, and lived experience (11, 29). Together, these perspectives suggest that resilience is not simply located within persons, but emerges through person–environment relationships.

Social support is one of the clearest examples of this relational logic. Classic stress-buffering theory proposed that social support protects individuals from the adverse effects of stress by providing emotional, informational, and material resources (31). Yet, in global crises, social support itself may be disrupted by displacement, bereavement, isolation, polarization, mobility restrictions, digital exclusion, or the erosion of community trust. Conversely, communities with stronger social cohesion, collective efficacy, shared values, and accessible resources may be better able to organize mutual aid, support vulnerable members, sustain meaning, and recover after disruption (10, 13, 22, 32). Social resilience therefore supplements rather than replaces individual coping: it provides the collective conditions that make adaptive coping possible.

This shift is especially important for public mental health. Individual-level interventions such as psychotherapy, pharmacological treatment, psychoeducation, stress management, and skills-based coping programs remain essential. However, they are insufficient when distress is generated or maintained by unsafe housing, climate exposure, political violence, institutional mistrust, unemployment, fragmented communities, or lack of access to care. In such contexts, effective mental health responses must also include community-based psychosocial support, strengthened primary care, social protection, culturally meaningful practices, trusted communication, participatory decision-making, and policies that reduce structural vulnerability (1–3, 9, 10, 13, 22).

Table 1 summarizes the conceptual shift proposed in this section. The aim is not to oppose individual coping and collective resilience, but to show that coping becomes insufficient when crisis-related distress is produced by social, territorial, institutional, ecological, and political conditions.

**Table 1:** From individual coping to collective resilience: a conceptual shift.

Dimension	Individual-centered resilience	Collective and geopsychiatric resilience
Main unit of analysis	The individual and their coping capacities	People embedded in families, communities, territories, institutions, and global systems
Understanding of coping	Cognitive, emotional, and behavioral strategies to manage stress	Shared practices that interpret threat, distribute support, preserve meaning, and mobilize resources
View of resilience	Capacity to maintain or regain functioning after adversity	Dynamic process shaped by social connection, resources, trust, culture, and territory
Source of vulnerability	Personal history, symptoms, coping style, psychiatric vulnerability, and perceived control	Unequal exposure to crisis, poverty, displacement, unsafe housing, institutional neglect, climate risk, violence, and lack of care
Main intervention target	Strengthen individual coping, emotion regulation, self-efficacy, and help-seeking	Strengthen communities, social protection, trusted communication, primary care, institutions, and structural prevention
Role of place	Background context or demographic variable	Active determinant of exposure, access, belonging, safety, mobility, and recovery
Role of institutions	Service providers for individuals in distress	Producers of trust, recognition, protection, access, and collective efficacy
Risk of misuse	Blaming individuals for not adapting sufficiently	Romanticizing community resilience while ignoring structural violence and institutional responsibility
Public mental health implication	Provide clinical care and individual coping support	Build environments in which people are not left to cope alone

A geopsychiatric approach helps conceptualize this broader field of action. If distress is shaped by territory, ecology, governance, mobility, culture, and institutional capacity, then resilience must also be understood across these levels. Individual coping remains one component, but not the unit around which the entire response should be organized. The question is not only whether individuals can regulate emotions or adapt to stress, but whether communities have the social, material, cultural, and political resources required to sustain mental health under conditions of crisis. This reframing does not deny personal agency; it protects it from being overburdened.

## 3 Emotional engagement as a public mental health process

Emotional engagement in global crises should not be understood only as an individual psychological reaction to external events. Although emotions are experienced by individuals, they are interpreted, communicated, shared, amplified, and regulated within social worlds. In crises, people do not simply feel fear, grief, anger, uncertainty, or hope in isolation; they encounter these emotions through families, communities, media narratives, political discourse, cultural memory, and institutional responses. The social sharing of emotion is therefore a central mechanism through which private distress becomes collectively meaningful and communities make sense of threat, loss, uncertainty, and recovery (19, 20, 33).

This collective dimension is especially relevant when crises are prolonged, ambiguous, or geographically dispersed. Pandemics, climate change, armed conflicts, forced migration, and political instability may generate emotional conditions that exceed the boundaries of direct exposure. People may experience distress not only because they personally suffer harm, but because they perceive shared vulnerability, witness the suffering of others, anticipate future losses, or feel that familiar social and ecological worlds are becoming unstable. Emotional engagement is therefore not merely a downstream effect of crisis; it is part of how crises are socially understood, politically framed, and publicly managed.

Risk perception research helps explain why the emotional meaning of a crisis may differ from its technical or epidemiological assessment. Slovic’s work showed that public responses to hazards are shaped not only by statistical probabilities, but also by perceived dread, uncertainty, catastrophic potential, controllability, and trust (34). Kasperson and colleagues’ social amplification of risk framework further showed that information processes, institutional responses, social groups, and media channels can intensify or attenuate public perceptions of risk (35). These insights are highly relevant to public mental health because emotional responses to crisis are shaped by how risk is communicated, who communicates it, and whether institutions are perceived as competent, honest, and protective.

A geopsychiatric perspective extends this logic by asking how risk perception and emotional engagement are distributed across territories and populations. The same crisis may have different emotional consequences depending on prior exposure to violence, climate vulnerability, poverty, migration history, political marginalization, cultural meanings, and access to care. Threat-oriented geopolitical rhetoric may further shape emotional climates by framing global events through instability, existential danger, competition, or loss of control (18). In such contexts, emotions are not only responses to material events; they are also responses to narratives about the future, institutional trust, and collective security.

Media and digital environments are central pathways through which emotional engagement becomes a public mental health issue. Research after collective traumatic events has shown that repeated exposure to distressing media coverage may be associated with acute stress and longer-term psychological and physical health effects, even among individuals not directly exposed to the event (36, 37). During pandemics and conflicts, continuous exposure to alarming headlines, images of suffering, uncertain expert commentary, and polarized online debate can create a persistent emotional background of vigilance and exhaustion; the COVID-19 pandemic also illustrated how media exposure and public communication may amplify anxiety, uncertainty, and risk-related behavior at the population level (38). Digital environments intensify this process because they collapse distance between local and global crises. They can support solidarity, rapid information exchange, tele-mental health, and community mobilization, but they can also amplify fear, misinformation, outrage, social comparison, and emotional contagion. From a public mental health perspective, the digital sphere is therefore one of the environments in which crisis emotions are produced, circulated, and regulated.

Climate-related emotions illustrate why emotional engagement should not automatically be pathologized. Ecological grief has been described as a legitimate response to climate change-related loss, including the loss of species, ecosystems, places, livelihoods, and anticipated futures (39). Eco-anxiety and climate anxiety likewise involve uncertainty, uncontrollability, anticipatory concern, and moral distress related to environmental degradation (40). These emotions may become clinically significant when they are persistent, impairing, or associated with hopelessness and functional disruption, but they may also reflect realistic awareness, ethical concern, and motivation for collective action (8, 39, 40). The public mental health challenge is therefore not to suppress crisis-related emotions, but to help communities transform fear, grief, and uncertainty into meaning, connection, agency, and protection.

Collective action research further shows that emotions can mobilize social responses when they are linked to shared identity, perceived injustice, collective efficacy, and a sense that action is possible (21). In emergencies, social identity processes can facilitate mutual aid, cooperation, and collective psychosocial resilience, especially when public communication reinforces solidarity rather than panic or blame (22). During the COVID-19 pandemic, social and behavioral science emphasized the importance of trust, leadership, social norms, communication, stress, coping, and collective behavior in shaping public responses (41). Guidance on managing pandemic transitions similarly highlighted the need to maintain trust, use clear language, anticipate misinformation, engage with media, and support resilience and self-efficacy (42). These principles are not only behavioral; they are emotional and relational.

For these reasons, emotional engagement should be considered a core dimension of public mental health preparedness. Crisis response cannot be reduced to risk communication, clinical service provision, or individual coping advice. It must also attend to the emotional infrastructures of communities: trust, belonging, shared meaning, cultural continuity, mutual recognition, credible leadership, and opportunities for participation. When these infrastructures are weakened, fear and uncertainty may fragment communities; when they are strengthened, emotions can become resources for solidarity, protection, and collective recovery.

A geopsychiatric model of collective resilience must therefore include emotional engagement as a mediating process between global crises and mental health outcomes. Crises generate exposure, loss, uncertainty, and disruption; communities interpret these experiences through social narratives, media systems, institutions, and cultural frameworks; and emotional responses influence coping, help-seeking, social cohesion, political participation, and trust. Understanding this dynamic is essential if public mental health is to move beyond advising individuals to cope and toward creating environments in which collective resilience can emerge.

## 4 Collective coping and community resilience

Collective coping refers to the ways in which groups, communities, institutions, and care systems respond to shared threats. It does not replace individual coping, but changes the level of analysis: rather than asking only how a person manages stress, it asks how social groups interpret adversity, distribute support, organize resources, preserve meaning, and sustain agency under disruption. In global crises, people often cope through relationships, shared routines, mutual aid, cultural practices, collective narratives, religious or spiritual resources, community organizations, advocacy networks, and local institutions. These forms of coping are part of the social infrastructure through which distress is contained, interpreted, and transformed (10, 11, 13, 22, 33).

Social capital is central to this process. It broadly refers to the networks, norms, trust, and reciprocity that facilitate cooperation within and across communities (43). Collective efficacy adds a related dimension: the shared belief that a group can organize and act to achieve common goals (44). In crisis contexts, social capital and collective efficacy shape who receives help, how information circulates, whether institutions are trusted, and whether communities can coordinate protective action. Disaster research has shown that social capital and social networks are key resources for survival, recovery, and community resilience, particularly when formal systems are delayed, overwhelmed, or distrusted (45). From a geopsychiatric perspective, these resources are not evenly distributed across territories; they are shaped by history, inequality, migration, violence, urban design, rurality, digital access, and institutional presence.

A useful distinction can be made between bonding, bridging, and linking forms of social capital. Bonding ties provide support within close groups, such as families, neighbors, faith communities, or peer networks. Bridging ties connect different groups and may reduce fragmentation across social, cultural, political, or economic boundaries. Linking ties connect communities with institutions, authorities, services, and decision-makers. All three are relevant to public mental health: bonding ties may provide immediate emotional and practical support, bridging ties may expand solidarity, and linking ties may improve access to care, resources, protection, and political voice (43, 45). When these forms are weak, fragmented, or captured by exclusionary dynamics, collective coping may become less effective or may reinforce inequality.

Community resilience builds on these processes but should not be understood as mere recovery to a previous state. In many crisis contexts, returning to the pre-crisis situation may be neither possible nor desirable, especially when that prior state was marked by inequality, violence, marginalization, institutional neglect, or environmental vulnerability. Community resilience therefore includes adaptation and transformation, not only restoration (10, 13, 32). It involves the capacity to protect vulnerable members, maintain social connection, preserve cultural meaning, reorganize care, learn from disruption, and address structural conditions that make future harm more likely.

This distinction is important because collective coping can take both adaptive and maladaptive forms. Communities may respond to crisis with solidarity, cooperation, and mutual protection, but also with scapegoating, denial, exclusion, rumor, stigma, or polarization. Social identity can facilitate mutual aid and psychosocial resilience when people perceive shared fate and inclusive group membership (22). However, when crisis narratives divide populations into deserving and undeserving groups, or when political communication intensifies blame and fear, collective emotions may undermine social cohesion rather than strengthen it (18, 21). Public mental health responses should therefore not romanticize community. Communities are powerful sites of care, but they are also shaped by conflict, hierarchy, exclusion, and unequal access to voice.

Community resilience interventions should therefore be participatory rather than imposed. Pfefferbaum and colleagues have argued that such interventions should use multihazard approaches, include community assessment, focus on engagement, consider both assets and needs, adhere to ethical principles, and encourage skill development (46). This is directly relevant to a geopsychiatric framework because communities differ in exposures, histories, infrastructures, cultural resources, and institutional relationships. Standardized resilience interventions designed without local participation may fail to address the actual sources of distress or overlook existing protective practices. Participatory processes, by contrast, can identify local strengths, trusted actors, barriers to care, and culturally meaningful pathways of recovery.

Mental health and psychosocial support frameworks in humanitarian settings already recognize the importance of multilayered, community-based responses. The Inter-Agency Standing Committee guidelines emphasize coordinated supports ranging from basic services and security to community and family supports, focused non-specialized supports, and specialized services (47). This layered model avoids reducing crisis response to clinical treatment alone and recognizes that distress may also be alleviated through safety, information, social reconnection, culturally appropriate support, and restoration of services. Evidence from humanitarian mental health and psychosocial support further highlights the need to link practice, funding, and research so that interventions are contextually appropriate, scalable, and responsive to local needs (48).

Community mental health interventions also show that promoting mental health and social equity requires multisectoral action. Interventions involving schools, primary care, housing, criminal justice systems, community organizations, peer supports, and social services can address mental health needs while modifying the conditions that produce vulnerability (49). Neighborhood research has similarly linked collective efficacy with social cohesion and willingness to intervene for the common good, reinforcing the public mental health relevance of community-level agency (50). In global crises, collective resilience is therefore strengthened not only by psychological support, but also by social policy, rights protection, inclusive governance, and material security.

Digital connectivity now modifies collective coping in complex ways. Online spaces can enable rapid mutual aid, emotional support, public health messaging, tele-mental health, crowdfunding, community organization, and transnational solidarity. They can also intensify misinformation, panic, surveillance, harassment, polarization, and emotional contagion. A geopsychiatric model must therefore treat the digital environment as part of the ecology of resilience. Digital spaces shape how people perceive threat, identify with others, access support, mobilize action, and evaluate institutional credibility (18, 41, 42).

Collective coping and community resilience are therefore best understood as dynamic, relational, and place-based processes. They emerge from the interaction between social networks, cultural meanings, institutions, material resources, emotional climates, and political conditions. They can reduce suffering when they promote safety, belonging, agency, trust, and access to care, but become fragile when communities are fragmented, under-resourced, stigmatized, or excluded from decision-making. For public mental health, the implication is clear: resilience cannot be prescribed to individuals as a private duty. It must be built collectively through social conditions that make coping possible.

## 5 Geopsychiatry as an integrative framework

Geopsychiatry provides a framework for understanding mental health as a phenomenon shaped by geography, ecology, political conditions, population movement, culture, institutions, and systems of care. Its contribution is not simply to add “place” as another variable to psychiatric thinking, but to examine how place is produced through social, environmental, historical, and political forces. In this sense, geopsychiatry bridges clinical psychiatry, public mental health, social epidemiology, environmental health, migration studies, disaster response, and policy. It asks not only what symptoms people experience, but also where suffering occurs, why certain populations are more exposed, how vulnerability is distributed, and which systems are capable of supporting recovery (7, 8, 13–18).

This integrative orientation is consistent with ecosocial theory, which emphasizes embodiment: the idea that social and ecological conditions become biologically and psychologically incorporated across time (51). From this perspective, mental health cannot be separated from the environments in which people live, work, move, and seek care. Heat exposure, unsafe housing, forced displacement, poverty, discrimination, political violence, and institutional neglect are not background factors. They are conditions through which stress, uncertainty, exclusion, and loss become embodied as distress, illness risk, functional impairment, or reduced well-being.

This shift is especially important because global crises often operate through structural violence. Farmer and colleagues described structural violence as social arrangements that put individuals and populations in harm’s way by constraining access to resources, safety, and care (52). In mental health, structural violence may appear as delayed treatment, untreated trauma, medication interruption, food insecurity, detention, precarious migration status, exposure to violence, or lack of political recognition. These conditions do not simply increase risk; they shape the meaning of distress and the feasibility of coping. Geopsychiatry makes these structures visible by linking psychiatric outcomes to the systems that organize exposure and protection.

Syndemic theory also helps clarify the value of a geopsychiatric framework, because it emphasizes that health problems cluster, interact, and worsen under harmful social conditions (53). Global crises rarely affect mental health through a single pathway. A conflict may produce trauma, displacement, poverty, family separation, interrupted education, health-system collapse, and vicarious media exposure. Climate change may generate heat stress, food insecurity, livelihood loss, migration pressure, ecological grief, and political conflict. Pandemics may combine infection risk, isolation, bereavement, economic insecurity, misinformation, and service disruption. A geopsychiatric model is useful precisely because it can account for interacting crises rather than treating each stressor as a separate exposure.

Migration illustrates this complexity. The UCL–Lancet Commission on Migration and Health emphasized that migration is shaped by political, economic, social, and environmental forces, and that migrant health depends on conditions before departure, during transit, at destination, and after return (54). From a geopsychiatric perspective, migration is not only a demographic event or a risk factor for selected disorders. It reorganizes identity, belonging, family structure, legal status, access to care, exposure to discrimination, and relationships with territory. Forced migration, climate-related displacement, and conflict-driven mobility may therefore affect mental health through trauma, uncertainty, loss of home, disrupted social networks, cultural dislocation, and institutional exclusion (5, 7, 54).

Urbanization and the built environment are also geopsychiatric concerns. Urban mental health is shaped by density, housing quality, green space, transportation, pollution, noise, violence, social cohesion, segregation, and access to services (55, 56). Cities may offer opportunity, diversity, specialized care, education, and social mobility, but they may also produce loneliness, overcrowding, inequality, environmental exposure, and fragmented social relations. Rural and remote areas may provide strong community ties and cultural continuity, but may also face limited mental health services, climate-sensitive livelihoods, poverty, stigma, and geographic isolation. A geopsychiatric approach therefore avoids assuming that urban or rural spaces are inherently protective or harmful; instead, it examines how specific territorial arrangements create risk or resilience.

Climate change provides one of the clearest examples of why psychiatry requires a geopsychiatric lens. Climate-related mental health outcomes may follow acute events such as floods, wildfires, storms, and heatwaves, but may also emerge through chronic environmental degradation, livelihood disruption, displacement, uncertainty, and anticipatory distress (6–8, 16, 57). These pathways are unevenly distributed, as are protective resources such as cooling, stable housing, insurance, healthcare, social support, political voice, and trusted institutions. Geopsychiatry therefore frames climate-related distress as both psychological and territorial.

Importantly, geopsychiatry does not replace individual-level psychiatry. Clinical diagnosis, psychotherapy, psychopharmacology, crisis care, and rehabilitation remain essential. Its contribution is to prevent these tools from becoming context-blind. A patient presenting with anxiety after forced displacement, depressive symptoms after crop loss, insomnia during political violence, or hopelessness in the face of climate disruption may need clinical care, but that care will be incomplete if it ignores the conditions producing and sustaining distress. Geopsychiatry therefore supports a both/and approach: individual suffering must be treated, while the structural, territorial, and institutional sources of suffering must also be recognized.

This approach has implications for public mental health systems. If global crises operate across ecological, social, political, and digital environments, then mental health responses must be multisectoral. Health systems need to coordinate with housing, education, social protection, emergency management, migration services, climate adaptation, human rights institutions, and community organizations (1–3, 47–49). A geopsychiatric framework can help identify where vulnerabilities cluster, which communities are underprotected, how services should be adapted to local conditions, and where prevention should be located before crises become clinical emergencies.

Geopsychiatry is therefore especially useful for reframing resilience. Individual resilience asks whether a person can adapt to stress. Collective resilience asks whether social groups can maintain connection, meaning, protection, and agency. Geopsychiatric resilience asks how territory, ecology, institutions, culture, mobility, digital systems, and political conditions enable or constrain both individual and collective adaptation. This broader lens is essential in an era when crises are not only psychological events, but also planetary, geopolitical, and social processes that reshape the conditions of mental life.

## 6 Discussion

### 6.1 Toward a geopsychiatric model of collective resilience in global crises

The central contribution of this Conceptual Analysis is the proposal that resilience in global crises should be understood not only as an individual psychological capacity, but as a multilevel geopsychiatric process. The preceding sections show that coping and resilience are shaped by emotional engagement, social networks, territory, institutions, ecology, culture, digital environments, and systems of care. We therefore propose a geopsychiatric model of collective resilience in global crises. This model does not replace clinical, community, or public health frameworks; rather, it organizes them within a broader structure that explains how global crises become mental health risks and how collective resilience may be strengthened across contexts.

The model begins from the premise that global crises affect mental health through interacting pathways rather than isolated exposures. A pandemic, heatwave, conflict, migration crisis, or episode of political instability may affect individuals directly, but it also reshapes family routines, community cohesion, institutional trust, access to care, media environments, livelihoods, and future expectations. These processes unfold across individual, relational, community, territorial, institutional, digital, and global levels. This multilevel organization is consistent with Bronfenbrenner’s ecological systems theory, which understands human development as occurring through dynamic interactions between individuals and nested, interconnected environments (58). These environments include immediate relationships and settings, interactions across those settings, institutions and services that indirectly influence development, and broader cultural, socioeconomic, and political structures. The later bioecological formulation further emphasized the interdependence of process, person, context, and time, recognizing that person–environment relationships evolve across the life course, social transitions, and historical periods (59).

The proposed geopsychiatric model builds on this ecological foundation while extending its application to contemporary global crises. It foregrounds territorial exposure, population mobility, institutional trust, geopolitical conditions, digital environments, environmental degradation, and planetary processes that may operate across and beyond conventional ecological levels. It also places collective resilience at the center of the framework, emphasizing that adaptation depends not only on interactions between individuals and their environments, but also on the capacity of communities, institutions, and systems to provide protection, resources, recognition, and opportunities for agency. This ecological interpretation also aligns with social determinants frameworks that locate health within broader layers of social, economic, cultural, and environmental conditions (3, 60).

At the individual level, crises may generate psychological distress, anxiety, depressive symptoms, grief, trauma-related reactions, sleep disturbance, hopelessness, substance use, and functional disruption (4–6, 8, 23–28). However, individual coping is only one layer within a wider ecology of supports and constraints. At the relational and community levels, the model emphasizes connectedness, social support, collective efficacy, social capital, shared identity, mutual aid, and community competence (9–11, 13, 22, 43–45). These processes influence how people interpret threat, access practical help, maintain belonging, and participate in collective action.

At the territorial level, the model focuses on place, exposure, mobility, and material infrastructure, including climate conditions, housing quality, urban or rural context, transport, green space, environmental degradation, borders, displacement routes, and access to services (7, 16, 32, 54–57). At the institutional level, it includes governance, health systems, social protection, education, emergency preparedness, human rights protections, migration policy, climate adaptation, and public communication. Institutions shape mental health by delivering services, but also by producing trust or distrust, recognition or abandonment, and protection or exposure (1–3, 41, 42, 47–49). Institutional trust is therefore not merely a political variable; it is a public mental health resource.

The digital and global levels complete the model. Contemporary crises are increasingly experienced through information ecosystems that may support tele-mental health, social support, public health messaging, community organization, and transnational solidarity, while also amplifying misinformation, threat narratives, emotional contagion, polarization, and exposure to distressing images (18, 36–38, 41, 42). At the global level, local mental health is situated within planetary and geopolitical systems. Climate change, armed conflict, forced migration, economic shocks, pandemics, and political instability interact with one another and may cluster syndemically under conditions of inequality, weak governance, environmental degradation, and social fragmentation (51–53, 61). The planetary health framework is relevant here because it links human health to the state of natural systems and emphasizes that environmental degradation can undermine the foundations of well-being (61).

Within this model, emotional engagement operates as a mediating process. Crises generate exposure, loss, threat, and uncertainty, but their mental health effects are shaped by how emotions are socially interpreted, shared, amplified, and mobilized (19, 20, 33–37, 39, 40). Fear may lead to avoidance, vigilance, stigma, or protective action; grief may lead to withdrawal, meaning-making, ritual, or solidarity; anger may lead to polarization or advocacy; and hope may support persistence, agency, and collective recovery. Emotional engagement therefore links perception, meaning, behavior, and social response.

**Figure 1:**
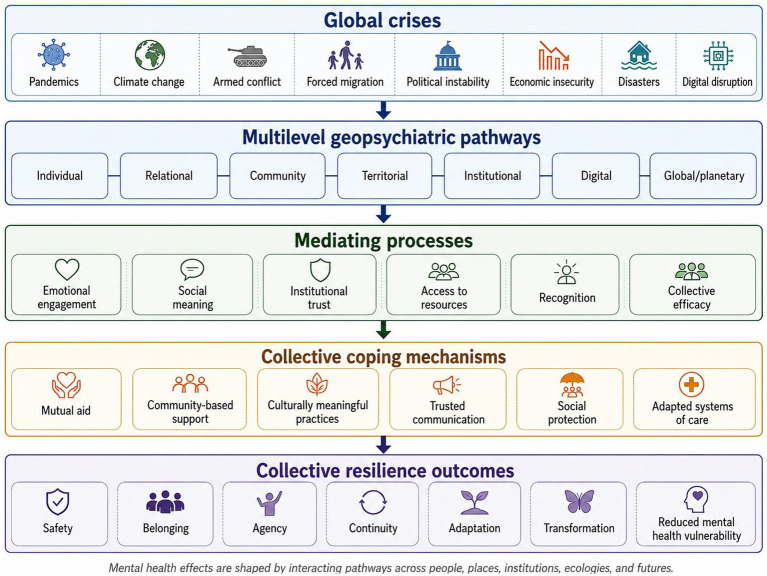
A geopsychiatric model of collective resilience in global crises. Global crises affect mental health through interacting individual, relational, community, territorial, institutional, digital, and planetary pathways. These pathways are mediated by emotional engagement, social meaning, institutional trust, access to resources, recognition, and collective efficacy. Collective coping mechanisms, including mutual aid, culturally meaningful practices, community-based support, trusted communication, social protection, and adapted systems of care, may strengthen collective resilience by promoting safety, belonging, agency, continuity, adaptation, and transformation.

Figure 1 summarizes this geopsychiatric model of collective resilience in global crises.

### 6.2 Public mental health, policy, and care implications

This model has several implications for public mental health. First, crisis-related mental health responses should not be limited to individual coping advice, psychoeducation, or clinical service delivery. These interventions remain necessary, but they are insufficient when distress is generated or maintained by unsafe housing, displacement, climate exposure, institutional mistrust, social fragmentation, unemployment, or lack of access to care. Public mental health strategies should therefore strengthen the conditions that make coping possible: safety, social connection, credible information, community participation, culturally meaningful support, access to services, and material protection.

Second, mental health systems should be integrated into broader crisis preparedness and response. Humanitarian mental health and psychosocial support frameworks already emphasize layered responses that include basic services and security, community and family supports, focused non-specialized supports, and specialized care (47, 48). A geopsychiatric approach extends this logic by connecting mental health planning with housing, education, migration services, social protection, emergency management, climate adaptation, human rights institutions, and digital governance (1–3, 49, 62). This is particularly important in low-resource settings, where mental health systems may be asked to respond to crisis-related distress without the social and institutional supports required to sustain recovery.

Third, public communication should be treated as a mental health intervention. In crises, communication does more than transmit information; it shapes risk perception, emotional engagement, institutional trust, and collective efficacy (34, 35, 41, 42). Communication that is inconsistent, stigmatizing, politically manipulative, or excessively alarmist may intensify distress and undermine cooperation. Conversely, clear, transparent, culturally sensitive, and solidarity-oriented communication can help communities interpret threat, reduce uncertainty, and mobilize protective action.

Fourth, digital environments should be incorporated into crisis mental health planning. Digital spaces are now part of the geography of mental health: they influence how people encounter crisis, share emotion, access support, mobilize action, and evaluate institutions. Public mental health responses should therefore promote digital literacy, responsible risk communication, access to tele-mental health, and strategies to reduce misinformation, harassment, and repeated exposure to distressing content.

Finally, the model suggests that resilience should be understood as a temporal process. Immediate responses may focus on safety, stabilization, information, and access to basic needs. Medium-term responses may require social reconnection, community-based psychosocial support, restoration of services, and adaptation of care pathways. Long-term resilience requires addressing structural vulnerability, rebuilding trust, strengthening health systems, preserving cultural continuity, and adapting institutions to recurrent crises (9, 10, 47, 48, 62). Resilience should therefore not be equated with rapid recovery alone. In some contexts, resilience may mean endurance; in others, adaptation; in others, transformation.

### 6.3 Limitations and future directions

This article has limitations. First, it is a conceptual analysis rather than a systematic review or empirical study. We do not estimate effect sizes, compare interventions, or test the proposed model with original data. Second, the model is intentionally broad, because it seeks to integrate clinical, community, territorial, institutional, digital, and planetary dimensions of crisis-related mental health. This breadth is useful for conceptual synthesis, but it also requires future operationalization. Third, the model may need adaptation across sociocultural, political, and economic contexts. What counts as collective resilience in one community may differ in another, especially where histories of colonization, conflict, migration, inequality, or institutional neglect shape the meaning of crisis and recovery.

Future research should test and refine the model empirically. Studies could examine how emotional engagement, institutional trust, territorial vulnerability, digital exposure, social capital, and collective efficacy interact to predict mental health outcomes during crises. Longitudinal and multilevel designs would be especially useful for distinguishing acute distress from longer-term adaptation or deterioration. Comparative studies across countries, cities, rural areas, displaced populations, and climate-vulnerable communities could clarify how geopsychiatric resilience varies by context. Future studies should also adopt intersectional approaches to examine how social positions, structural disadvantage, territorial exposure, and institutional exclusion interact in shaping crisis-related distress and collective resilience. Future work should also develop measures of collective and geopsychiatric resilience that move beyond individual trait-based scales and include community, institutional, territorial, and digital dimensions.

## 7 Conclusion

Global crises are transforming the conditions under which mental health is produced and protected. Pandemics, climate change, armed conflict, forced migration, political instability, economic insecurity, and digital disruption do not affect mental health only by exposing individuals to stress. They reshape the social, territorial, institutional, ecological, digital, and symbolic environments in which people cope, seek support, trust institutions, access care, and imagine the future. In this context, resilience cannot be adequately understood as a private capacity to withstand adversity. It must also be understood as a collective and contextual process shaped by the resources, relationships, infrastructures, narratives, and institutions that make adaptation possible.

A geopsychiatric model of collective resilience reframes the central question of crisis mental health. Instead of asking only how individuals can cope with global crises, it asks how communities, territories, institutions, and systems can be organized so that people are not left to cope alone. This shift does not diminish the importance of clinical care, individual agency, or psychological support. Rather, it situates them within the broader environments that determine whether coping is feasible, whether distress is recognized, and whether recovery can be sustained.

Collective resilience is therefore not a slogan or a personal virtue. It is a geopsychiatric condition: a product of relationships between people, places, institutions, ecologies, and futures. Public mental health responses to global crises should therefore move beyond exhortations to individual resilience and toward the creation of social, territorial, institutional, and digital conditions that protect mental health, strengthen trust, support collective coping, and enable communities to adapt without being abandoned to crisis.
